# Effects of Exercise on Skeletal Muscle Pathophysiology in Huntington’s Disease

**DOI:** 10.3390/jfmk7020040

**Published:** 2022-05-11

**Authors:** Bruno Trovato, Benedetta Magrì, Alessandro Castorina, Grazia Maugeri, Velia D’Agata, Giuseppe Musumeci

**Affiliations:** 1 Department of Biomedical and Biotechnological Sciences, Human, Histology and Movement Science Section, University of Catania, Via S. Sofia n°87, 95123 Catania, Italy; brunotrovato94@gmail.com (B.T.); benedetta89@hotmail.it (B.M.); graziamaugeri@unict.it (G.M.); vdagata@unict.it (V.D.); 2 Laboratory of Cellular and Molecular Neuroscience (LCMN), School of Life Sciences, Faculty of Science, University of Technology Sydney, Sydney, NSW 2007, Australia; alessandro.castorina@uts.edu.au; 3 Laboratory of Neural Structure and Function, School of Medical Science (Neuroscience), University of Sydney, Sydney, NSW 2006, Australia; 4 Research Center on Motor Activities (CRAM), University of Catania, Via S. Sofia n°97, 95123 Catania, Italy

**Keywords:** Huntington’s disease, exercise, skeletal muscles, mouse models, rehabilitation, motor function

## Abstract

Huntington’s disease (HD) is a rare, hereditary, and progressive neurodegenerative disease, characterized by involuntary choreatic movements with cognitive and behavioral disturbances. In order to mitigate impairments in motor function, physical exercise was integrated in HD rehabilitative interventions, showing to be a powerful tool to ameliorate the quality of life of HD-affected patients. This review aims to describe the effects of physical exercise on HD-related skeletal muscle disorders in both murine and human models. We performed a literature search using PubMed, Scopus, and Web of Science databases on the role of physical activity in mouse models of HD and human patients. Fifteen publications fulfilled the criteria and were included in the review. Studies performed on mouse models showed a controversial role played by exercise, whereas in HD-affected patients, physical activity appeared to have positive effects on gait, motor function, UHDMRS scale, cognitive function, quality of life, postural stability, total body mass, fatty acid oxidative capacity, and VO2 max. Physical activity seems to be feasible, safe, and effective for HD patients. However, further studies with longer follow-up and larger cohorts of patients will be needed to draw firm conclusions on the positive effects of exercise for HD patients.

## 1. Introduction

Huntington’s disease (HD) is a hereditary neurodegenerative disorder characterized by progressive motor dysfunction, psychiatry disturbances, and cognitive deficit [1,2]. The genetic basis of the disease is an abnormal expansion of the cytosine-adenine-guanine (CAG) trinucleotide repeat in the IT15 gene on chromosome 4 [3]. Nowadays, a genetic test can identify individuals at risk of inheriting the expanded CAG nucleotide before the onset of clinical symptoms. The mean age of onset is around 40 years, and the disease progression leads to death in 15 to 20 years [4]. Biological evidence indicates that an increasing number of CAG repeats promote the functioning and survival of brain neurons which are crucial for embryonic development [5]. In the healthy population, CAG repeat lengths in the HTT gene vary significantly from 6 to 36. An expansion above 39 CAG repeats causes the manifestation of the pathology [6]. This mutation leads to an unusually long expansion of the polyglutamine tract in the protein that causes toxicity, subsequent dysfunction, and death of the striatal and cortical neurons [7]. The complex mechanisms of the pathophysiology behind Huntington’s disease are not fully understood yet. In a recent study on individuals that carry the mutation that causes HD, it was found that CAG repeats of 37 to 42 were associated with higher cognitive skill development, while longer repeats were associated with lower skill levels [8]. When the pathology manifests itself, the most characteristic neuropathological abnormalities are neuronal loss in the basal ganglia and cerebral cortex [9]. The neurological degeneration leads to psychiatric symptoms like apathy, depression, irritability, aggressive behavior, anxiety, and also cognitive symptoms that affect attention, memory, and language [10]. The impact of cortical degeneration and dysfunction also give a significant contribution to impairments in motor functions [11,12]. Motor abnormalities include involuntary movements such as chorea and dystonia as well as disturbances such as bradykinesia [13,14]. Even if chorea is prominent in the early stages of the disease, later progressive bradykinesia, rigidity, and incoordination become functionally more disabling [15]. Moreover, many patients often have substantial cognitive or behavioral disturbances before the onset of motor symptoms [16]. Another symptom that leads to the damaging of motor functions is dystonia occurring in more than 90% of HD [17]. Choreatic symptoms decrease in later stages of HD, instead, dystonia tends to increase with the progression of the disease, probably due to direct pathway dysfunction [18].

**Figure 1:**
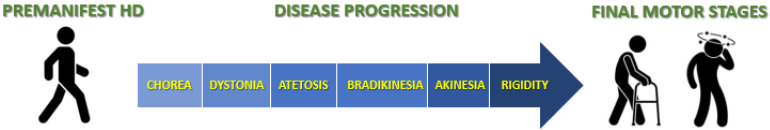
Motor symptoms related to disease progression.

Huntingtin (HTT) protein is widely distributed in a large variety of tissues, including brain, heart, skeletal muscles, kidneys, and liver [19]. Many studies on HD throughout the years have investigated the role of HTT protein in the brain but, recently, the importance of understanding the molecular mechanisms that lead to a deterioration of the skeletal muscles is growing. The pathogenetic mechanisms involved in muscle dysfunction are not fully understood yet. However, the pathological effects of HD on skeletal muscles have been demonstrated both in animal models and humans [20,21,22]. Studies performed on muscle of HD transgenic mice and on muscle cell cultures from HD patients showed mitochondrial dysfunction, decreased levels of ATP, oxidative stress, and inflammation [23,24]. Braubach et al. [25] found that the skeletal muscles of HD mice exhibit severely disturbed Ca^2+^ homeostasis with a significant reduction of Ca^2+^ entry, release, and removal. Romer et al. [26], found that the t-tubule network of HD mice was intact but the diameter of the individual t-tubules was reduced, causing disrupted Ca^2+^ signaling that may explain the symptoms of weakness and fatigue in HD. In a study evaluating the symptomatic HD patients with a 31P magnetic resonance spectroscopy, a reduction in phosphocreatine to inorganic phosphate ratio at rest was found [27]. The authors reported also that ATP/phosphocreatine and inorganic phosphate levels in muscles were significantly reduced in HD patients compared to control. Furthermore, in another study, Ciammola et al. [28], showed that pre-symptomatic HD subjects have a lower anaerobic threshold and increased level of plasma lactate compared to control. Gehrig et al. [29] showed that the percentage of type 1 muscles fibers is in proportion, significantly higher in HD patients compared to control. Moreover, the mitochondrial respiratory capacity specific to complex I and the capacity of maximal oxidative phosphorylation were marginally lower in HD patients [30]. The lower percentage of type 2 fibers found in HD patients could help the interpretation of the findings by Busse et al. [30], whose HD patients exhibited nearly half the isometric strength of healthy control when different muscle groups were evaluated with a handheld dynamometer. At a clinical level, the most typical motor symptom of HD is chorea, which is characterized by abnormal involuntary movement and brief, irregular contractions that appear to flow from one muscle to the other. Moreover, chorea often comes along with athetosis that causes also twisting and writhing movements [31]. During the progression of the disease, dystonia also occurs and the facial muscles are affected until, in later stages, dysarthria and dysphagia become a serious issue. Furthermore, HD patients also develop hypokinesia, bradykinesia, rigidity, and akinesia [32], (Figure 1). The impairments affecting the musculoskeletal systems also lead to gait and balance impairments in HD patients. Premanifest HD patients show slower gait velocity and cadence, shorter stride length, and poor dynamic balance control, compared to healthy people [33]. Patients with manifest HD have even worse gait cadence and velocity compared to premanifest HD and increased amplitude and velocity of mediolateral trunk’s sway. They also manifest a wider base of support, poor balance, and difficulties in dual-tasking [33].

The management of HD is currently based on symptomatic treatment, and widely directed at the chorea and neurobehavioral problems [34]. However, none of these treatments has a long-term disease-modifying effect. Physical exercise in the treatment of HD-affected patients was investigated throughout the years [35]. This review aims to describe HD-related skeletal muscles disturbances and highlight the effect of physical exercise and multidisciplinary rehabilitation on motor functions in persons with HD.

## 2. Materials and Methods

In this narrative review, we provided an overview on the impact of HD on the musculoskeletal system and on the effects of physical activity both in mouse models and human patients. Keywords for literature included “Huntington’s disease”, “muscle HD pathophysiology”; “muscle wasting HD”, “mouse models HD”, “Huntington mouse exercise” “motor function HD”, “gait HD”, “physical exercise HD”, “rehabilitation HD”, “physical therapy HD”. The searches were limited to studies published in English language that included mouse models or humans HD patients. The study design included narrative, systematic reviews, and original articles. We started the literature search from July 2021 to January 2022 on PubMed, Scopus, and Web of science databases. Twenty-one sources met the eligibility criteria, considered appropriate for the purpose of the review. All the included studies were original article, presented in Table 1. Considering the great variability present in physical exercise protocols for the treatment of HD, we evaluated that a narrative review was the most appropriate form for our study.

## 3. The Effects of Physical Activity in Mouse Models and Patients with HD

### 3.1. The Effects of Physical Activity in HD Rodent Models

Studies throughout the years have shown the positive association between physical exercise and a lower risk of developing neurodegenerative diseases [57,58]. In the mouse models, running improves hippocampal neurogenesis, spine density, vascularization, neurotrophins levels, learning, and long-term potentiation [59,60]. Moreover, acute treadmill exercise induced in mouse skeletal muscle, the formation of new intracellular junctions, known as calcium entry units (CEUs). CEUs, containing proteins used to promote Ca^2+^ entry and storage, represent an important element of muscle adaptation during fatigue [61]. Exercise-induced CEUs, increased Orai1-dependent Ca^2+^ entry to favor myoplasmic Ca^2+^ dynamics, reducing muscle force decline during sustained activation [62,63]. Noteworthy, exercise supports the production and secretion of myokines by skeletal muscles [64], such as BDNF, which promotes HD mice’s motor functions and survival rate, by counteracting brain atrophy [65,66]. However, the effects of physical exercise on HD animal models reported controversial data. In HD rodent models, treadmill exercise improved motor coordination, spatial learning, and short-term memory [50,51]. On the contrary, Wood et al. [52] showed that physical exercise on the rotarod did not significantly improve motor coordination of R6/2 mice, although it induced no deleterious effects. Exercise on a motorized treadmill and wheel-running improved cognition and prevented depressive-like behaviors [53,54,55]. Moreover, voluntary exercise postponed the onset of pathology, by ameliorating cognitive ability [67]. Exercise improved hindlimb clasping symptoms and prevented the alteration in mitochondrial content and function occurring in the late stage of HD [68]. In accord, Caldwell et al. showed that treadmill training was effective in enhancing mitochondrial function in the CAG140 KI HD mouse model, resulting in improved motor performance [49]. Moreover, Van Dellen et colleagues demonstrated that wheel running from a juvenile age can delay the onset of some, but not all, motor deficits in a mouse model of HD [37].

On the other hand, it has been shown that running accelerates the age of onset and increases the severity of HD symptoms in N171-82Q transgenic HD mouse models [38]. Moreover, endurance training was detrimental for HD mice, inducing in skeletal muscle the activation of AMPK [56], whose increase is known to promote neuronal death by reducing HD mice’s lifespan [69].

## 3.2. The Effects of Physical Activity in HD Patients

**Figure 2:**
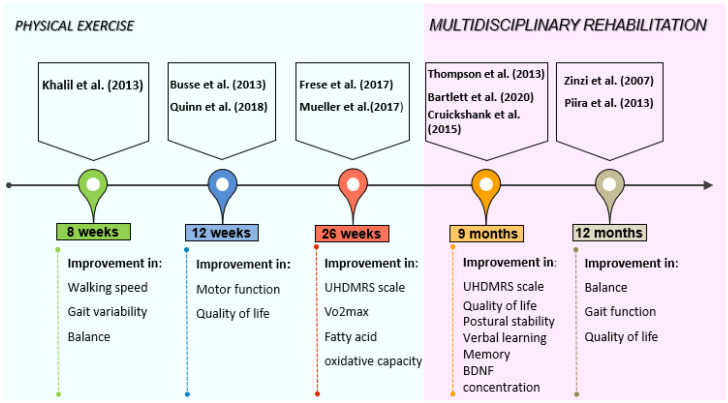
Intriguing timeline of physical exercise and multidisciplinary rehabilitation interventions with their relative outcomes.

Nowadays the negative impact of HD on motor functions and cognition in humans is well-known. The evaluation of motor function is based on the UHDRS-TMS scale, which permits the evaluation of movements, gait, hand movements, dystonia, and chorea [70]. There are two phases in HD regarding the motor functions, the hyperkinetic one, with chorea, and the hypokinetic one, characterized by dystonia, bradykinesia, gait, and balance perturbations [71]. The employment of physical activity intervention programs to improve motor functions in HD patients seems to be effective, reducing the effects of the natural disease progression [72] (Figure 2). Table 2 gives an overview description of physical activity interventions. Different authors investigated the integration of physical activity in multidisciplinary rehabilitation to ameliorate the quality of life in Huntington’s disease patients [36,39,40,43,44,48]. A study performed on 40 HD-affected patients, showed that a rehabilitation program that includes respiratory exercises, speech therapy, physical/occupational therapy, and cognitive rehabilitation can help the maintenance of functional and motor performance in patients with early to moderate HD [36]. An improvement in UHDRS-TMS score and quality of life, along with a reduction of motor and postural stability deterioration, was found by Thompson et al. [39], in early to middle stage of HD patients which performed nine months of multidisciplinary rehabilitation. The protocol used by the authors consisted of 9 months of 40 min aerobic training once a week supervised in an exercise clinic, occupational therapy 1 h every two weeks for six months, and a tailored, home-based, self-monitored exercise program three times per week for 6 months. Piira et al. [40], showed that a multidisciplinary approach, comprehending physical exercise, social activities, and group/teaching sessions, is associated with improved balance, gait function, and quality of life in patients with early to middle stage HD. The duration of the intervention was one-year with 3 admissions of 3 weeks each, and 5 days of evaluation approximately 3 months after the last rehabilitation admission. The patients underwent 8 h of activities including physiotherapy, occupational therapy, speech therapy, training in gym/swimming pool, and group discussions. Cruickshank et al. [44], employed a 9-months program of supervised clinical exercise 1 time per week, self-directed home-based exercises three times per week, and occupational therapy once every 2 weeks. The supervised exercise program consisted of 1 h of aerobic and resistance training while the home-based program was focused on strengthening and fine motor exercises for 1 h. The findings from this study showed that multidisciplinary rehabilitation can give positive results in terms of increasing grey matter volume in the right caudate and both sides of the dorsolateral prefrontal cortex, leading to an improvement in verbal learning and memory. The positive effects of multidisciplinary rehabilitation with physical exercise on brain structure for Huntington’s disease patients were also seen by Bartlett et al. [48]. In their study, 18 HD patients (10 premanifest and 8 prodromal) underwent 9 months of aerobic and resistance training two times per week, bilingual exercise, dual-task training one time per week for 1 h, computerized cognitive training three times per week for 1 h, and social activities. The authors reported that the intervention groups showed significantly less right hypothalamic grey matter volume loss than the control group and maintained higher concentrations of brain-derived neurotrophic factors, indicating that this kind of intervention can be beneficial for HD patients.

The effects of physical exercise alone were investigated by several authors [41,42,45,46,47]. An exercise protocol of 12 weeks of walking and cycling aerobic training (55–75% of predicted HRmax) and resistance exercises with leg press, leg extension, lat pulldown, hamstring curl, calf raises (2 × 8–12 reps at 60–70% of 1RM) maintained motor function stable and gave improvements on the quality of life [41]. In a randomized controlled pilot trial of Khalil et al. [42], on 25 early to mid-stage HD patients, a home-based program of 8 weeks of gradual progressive walking exercise three times per week resulted in an improvement of walking speed, gait variability, and balance measured with the Berg balance scale. Improvements in motor function measured with UHDRS motor score, and fitness measured with predicted VO2max, were found by Quinn et al. [45], in HD-affected patients that underwent 12 weeks of 30 min cycling training (65–85% of age predicted HRmax) and 10–15 min of strengthening exercises like chair stand, seated wood chop, plank, and chair lunges (2 × 10–12 reps). The study of Frese et al. [46], demonstrated the feasibility of aerobic training, high-intensity interval training (HIIT), and endurance training for HD patients. The intervention from the authors consisted of 10 weeks of 30 min cycling (65%VO2peak) 3 times per week, 8 weeks of HIIT (4 × 4 min at 90–95% of HRpeak with 3 min low-intensity rest intervals at 70% of HRpeak) three times per week, and 6 final weeks of endurance training three times per week [46]. This 26-weeks program resulted in an improvement of VO2peak, peak cycling power, and cycling time to exhaustion. Moreover, the UHDRS motor score remained stable indicating that the exercise could be beneficial for these patients. Another study of Mueller et al. [47], with the same protocol showed improvement of citrate synthase, complex III + V activity, and fatty acid oxidative capacity. Based on these studies, physical exercise exerted beneficial effects on HD-affected patients. However, all exercise training interventions have to be tailor-made, in terms of frequency, intensity, and specificity and accompanied by frequent assessments to avoid the acceleration and/or worsening of symptoms [72,73]. In fact, excessive training performed by a marathon runner provoked progressive myopathy many years before the first signs of chorea were detected [74].

The promising results from these studies indicate that exercise may be beneficial and feasible for HD patients and can enter the management of HD whether included in a multidisciplinary rehabilitation approach or alone.

## 4. Limitations

The current review presents different limitations: (1) the included studies are based on a small number of patients, with different disease stages and heterogeneity of disability. Works performed on larger cohorts might clarify the individual alterations in variables; (2) the differences in the training protocol used and the lack of control groups in some of these studies; (3) the multidisciplinary approach used in some works, which does not allow us to say with certainty that the positive effects are mainly due to physical exercise.

## 5. Conclusions

Several studies over the past few decades investigated how HD affects motor and cognitive functions. The effects of physical activity interventions in both mouse models and humans were also studied. To date, the treatment of HD relies mainly on pharmaceutical interventions to reduce the symptoms. However, recent studies are highlighting the positive effects that structured programs of physical exercise and multidisciplinary rehabilitation have on HD patients’ cognitive, motor functions, and quality of life. To the best of our knowledge, this is the first narrative review that summarizes the effects of HD on skeletal muscles and the positive role played by physical exercise and multidisciplinary rehabilitation. A major part of the reported studies sustains the beneficial role of physical activity for HD patients indicating that it should be prescribed, when possible, to ameliorate their quality of life. However, the exercise protocol has to be tailor-made to avoid the acceleration and/or worsening of symptoms.

## Figures and Tables

**Table 1:** Characteristics of included studies.

Authors/Year	Study Design	Model	Sample Size	Intervention	Result	Conclusion
**Zinzi et al. (2007)** [36]	Pilot clinical trials	Humans	n = 40	8 h a day for 5 days and 4 h a day for one day per week, repeated three times afor 1 year of physical, occupational and speech therapy, cognitive rehabilitation and respiratory exercises	Significant improvement of motor performance and activities of day living and maintaining of cognitive function	Intensive rehabilitation treatments may have a positive effest on motor and functional performance in patients with Huntington’s disease
**Van Dellen et al. (2008)** [37]	Mouse general health and behavioral assessment	Mice	n/a	Voluntary wheel running exercise from juvenile age (4 weeks) to adulthood (9 months). Open field and rotarod test at 5 months of age	Motor deficts on rotarod test delayed by wheel running as well as rear-paw clasping. Enviormental enrichment and wheel running decreased the abnormal locomotor activities	Voluntary wheel running started before the symptomatic stage of the disease can delay the onset of some motor deficits in HD mice
**Potter et al. (2010)** [38]	Mouse general health and behavioral assessment	Mice	n = 54	Voluntary running exercise from an age of 44 days to an age of 113 days. Morris water maze test at 88 days old, rotarod test at 100 days old and open field test at 103 days old	Running exercise worsened the HD motor deficits and accelerated its onset	Running is not effective in delaying HD symptoms. Exercise is not beneficial and may have a negative effect in the mouse model analyzed
**Thompson et al. (2013)** [39]	Randomized controlled trials	Humans	n = 20	Supervised group sessions of 9 months, once per week; 5 min warm-up, 10 min aerobic exercise, 40 min resistance exercise, 5 min cool-down. 6 months of home-based exercise 3 times per week. Occupational therapy for 1 h fortnight, for 6 months	Reduction of the loss of postural and dynamic stability; mild improvement in quality of life, depression and cognition; significant improvement in fat-free mass and strength	Multidisciplinary rehabilitation program is feasible and well tollerated in early to middle stage HD patients. Patients also reported therapeutic benefits
**Piira et al. (2013)** [40]	Prospective intervention study	Humans	n = 37	Physiotherapy, occupational and speech therapy, gym and/or swimming training, 8 h 5 days per week for 1 year.	Significant gains in balance, gait, activities of day living, quality of life, anxiety and depression	Multidisciplinary rehabilitation improved balance, gait function, and quality of life
**Busse et al. (2013)** [41]	Randomized feasibility study	Humans	n = 31	12 weeks of walking and cycling aerobic training at 55–75% of predicted HRmax; resistance exercises for lower limbs (2× 8–12 reps at 60–70% of 1 RM)	Moderate effect sizes showed benefits for cognitive and walking measures.	A structured exercise intervention gives improvements in motor function and quality of life in HD patients
**Khalil et al. (2013)** [42]	Randomized controlled pilot study	Humans	n = 25	home-based exercise for 8 weeks consisting of gradual progressive walking exercise 3 times per week	Walking speed and gait variability improvement with large effect sizes. Balance, funtion and level of phisical activity also had a significant improvement	Home based exercise are feasible, beneficial and safe for HD mid-stage patients
**Piira et al. (2014)** [43]	Prospective intervention study	Humans	n = 10	Physiotherapy occupational and speech therapy, gym and/or swimming training, 8 h five days per week for two years	Non significant decline of gait, balance and cognitive measures. Not significant increasing for quality of life, activities of day living and motor function	Intensive multidisciplinary rehabilitation is well tollerated among midlle stage HD patients.
**Cruickshawk et al. (2015)** [44]	Exploratory study	Humans	n = 15	1 h of aerobic and resistance training per week in clinic; 1 h of home based exercises 3 times per week; occupational therapy once every 2 weeks	Significant volumetric grey matter improvement and significant increasing of verbal learning and memory	Multidisciplinary rehabilitation may have a positive impacts on gray matter changes and cognitive functions in HD patients.
**Quinn et al. (2016)** [45]	Randomized controlled trial	Humans	n = 32	12 weeks of 30 min cycling training (65–85% of age predicted HRmax) and 10–15 min of strengthening exercises (2× 10–12 reps)	Improvement in VO_2_max, general fitness and motor function	A short-term exercise progam is safe, feasible and may be beneficial for midlle stage HD patients
**Frese et al. (2017)** [46]	Clinical trials	Humans	n = 24	10 weeks of 30 min cycling (65%VO2peak) 3 times per week, 8 weeks of HIIT (4 × 4 min at 90–95% of HRpeak with 3 min low-intensity rest intervals at 70% of HRpeak) 3 times per week and endurance training 3 times per week	Motor deficit stabilization, VO_2_max significant improvement	Specified exercise programs may induce therapeutic beneficial effects in HD patients
**Mueller et al. (2017)** [47]	Clinical trials	Humans	n = 24	10 weeks of 30 min cycling (65%VO2peak) 3 times per week, 8 weeks of HIIT (4 × 4 min at 90–95% of HRpeak with 3 min low-intensity rest intervals at 70% of HRpeak) 3 times per week and endurance training 3 times per week	Increasing in the activity of citrate synthase, complex III, complex V and succinate cytochrome c reductase	HD patients could benefit from an endurance training program in terms of delaying the progressive muscular dysfunction. The training program was also safe and feasible.
**Bartlett et al. (2020)** [48]	Pilot clinical trials	Humans	n = 29	9 months of aerobic and resistance training 2 times per week, bilingual exercise, dual-task training 1 time per week for 1-h, computerized cognitive training 3 times per week for 1 h and social activities.	Maintanance of serum BDNF levels, decreasing of cortisol and melatonin concentration	A program of multidisciplinary rehabilitation may be useful for maintaining peripheral BDNF levels and decreasing the hypothalamic volume loss in preclinical HD individuals
**Caldwell et al. (2020)** [49]	Mouse general health and behavioral assessment	Mice	n = 16	Running at a speed of 8.0 m/min for 40 min for first week; starting from second week exercise running at 10.0 ± 1.5 m/min 3 times per week for a 12-week period. Final month of exercise with running speed set at 20 ± 1.5 m/min	Treadmill exercise resulted in improved mitochondrial oxidative phosphorylation complex activity. Improvement were also registered fot glycolisys, pyruvate deidrogenase and carboxylase activity	Treadmill exercise may be beneficial for motor behavior thanks to reverisng deficits in mitochondrial function in a rodent model of HD
**E.S. Ji et al. 2015** [50]	Mouse general health and behavioral assessment	Mice	n = 40	30 min of treadmill running once a day for 14 days; running speed set at 2 m/min for the first 5 min, then at 5 m/min for the next 5 min and at 8 m/min for the last 20 min of exercise	Treadmill running exercise rescued motor coordination and suppressed caspase-3 expression	Running exercise could be beneficial in improving quinolinic acid-induced loss of spatial learning ability and coordination in HD mouse model
**Kim et al. 2015** [51]	Mouse general health and behavioral assessment	Mice	n = 40	30 min of treadmill running once a day for 14 days; running speed set at 2 m/min for the first 5 min, then at 5 m/min for the next 5 min and at 8 m/min for the last 20 min of exercise	Treadmill running exercise enhanced the production of neurotrophic factors in the brain and ameliorated memory and learnig ability	Treadmill exercise influences positively the cell proliferation in the hippocampal dentate gyrus by ameliorating the BDNF expression in HD rats; hence, treadmill exercise has beneficial effects HD symptoms
**Wood et al. 2011** [52]	Mouse general health and behavioral assessment	Mice	n = 128	11 days of training in Lashley III maze, then rotarod training for 1 day; lastly, 14 days of training in Lashley III maze again	There were no significant improvement in mice performance after training	Physical exercise on the rotarod did not significantly improve motor coordination of R6/2 mice, but it did not induced deleterious effects
**Renoir et al. 2012** [53]	Mouse general health and behavioral assessment	Mice	n = 140	4 weeks of voluntary wheel running exercise	Wheel running exercise and chronic setraline treatment prevent depressive like behaviours by correcting the 5-HT_1A_ autoreceptor dysfunction	Wheel-running exercise improved cognition and prevented depressive-like behaviours in R6/1 HD mice
**Stefanko et al. 2017** [54]	Mouse general health and behavioral assessment	Mice	n = 320	Running at a speed of 8.0 m/min for 40 min for first week; starting from second week exercise running at 10.0 ± 1.5 m/min 3 times per week for 6 months. Final month of exercise with running speed set at 20 ± 1.5 m/min	CAG140 KI mouse did not show significant worsening in performace at the rotarod test and forced swimmig test compared to wild type animals	A long term training program of running exercise is effective in delaying the onset of depression like behaviors in CAG140 KI mouse model of HD when started before the onset of motor symtoms
**Harrison et al. 2013** [55]	Mouse general health and behavioral assessment	Mice	n = 67	Running wheel exercise 14 h per day, for five days for 22 weeks. Behavioural testing every for weeks	Wheel running produced some benefit on stride length and reduction of striatal neuronal cell loss	Chronic wheel running exercise enhance cognitive funtion, reduce striatal cell loss in in the R6/1 HD mouse indicating that exercise may be benficial in HD
**Corrochano et al. 2018** [56]	Mouse general health and behavioral assessment	Mice	n/a	Forced endurance training protocol consiting in 30 min of rotarod set at 15 rpm, 5 day/week. Mice had also the oppurtunity to pratice voluntary running exercise in their cages	Endurance training was detrimental for HD mice, inducing the activation of AMPK in skeletal muscles	Physical activity that causes an high energy demands should be proposed to HD patients with caution

**Table 2:** Overview description of physical activity interventions. CG: control group; IG: intervention group; n/a= not available.

Authors	Participants Characteristics	Intervention Programs	Measured Outcome
**Zinzi et al. (2007)** [36]	n = 40 (M = 17; F = 23) age = 52.0 (3.3) CG = n/a	Respiratory exercise, speech therapy, physical therapy, occupational therapy, cognitive rehabilitation exercise	Balance, gait, depression, cognitive status, activities of day living
**Thompson et al. (2013)** [39]	n = 20 (M = n/a; F = n/a) age CG = 53.8 (2.9) age IG = 52.2 (2.9)	Aerobic exercise, resistance exercise, home-based occupational therapy	Motor function, cognition, body composition, postural stability, quality of life
**Piira et al. (2013)** [40]	n = 37 (M = 18; F = 19) age = 52,4 (13.1) CG = n/a	Physiotherapy, occupational and speech therapy, gym/swimming exercises, group discussions	Motor function, quality of life, cognitive function, depression/anxiety
**Busse et al. (2013)** [41]	n = 31 (M = 16; F = 15) age CG = 47.4 (9.5) age IG = 53.3 (12.5)	Aerobic training, resistance exercise	Motor function, quality of life
**Khalil et al. (2013)** [42]	n= 25 (M = n/a; F = n/a) age CG = 51.3 (16.9) age IG = 54.2 (9.9)	Resistance exercise, balance exercise.	Gait, balance, quality of life
**Cruickshawk et al. (2015)** [44]	n= 15 (M = 8; F = 7) age = 52.5 (6.6) CG = n/a	Home-based aerobic and resistance exercises, occupational therapy	Grey matter volume, verbal learning, memory
**Quinn et al. (2016)** [45]	n= 32 (M = 16; F = 16) age CG = 51.0 (17.0) age IG = 53.0 (11.0)	Aerobic training, resistance exercise	Motor function, fitness, cognition
**Frese et al. (2017)** [46]	n= 24 (M = 24; F = 0) age CG = 49.1 (6.8) age IG = 54.8 (7.1)	Aerobic and endurance training, high-intensity interval training	Motor function, dementia, cardiovascular performance
**Mueller et al. (2017)** [47]	n= 24 (M = 24; F = 0) age CG = 49.7 (6,8) age IG = 53.2 (8.8)	Aerobic and endurance training, high-intensity interval training	Mitochondrial function
**Bartlett et al. (2020)** [48]	n= 29 (M = 10; F= 19) age CG = 50.55 (9.49) age IG = 40.89 (11.73)	Aerobic exercise, resistance exercise, dual-task, bilingual exercise, cognitive training	Grey matter volume, BDNF concentration

## Data Availability

Not applicable.
